# Risk Factors for Macrosomia in Multipara: A Multi-Center Retrospective Study

**DOI:** 10.3390/children9070935

**Published:** 2022-06-22

**Authors:** Juan Juan, Yumei Wei, Geng Song, Rina Su, Xu Chen, Ruiqin Shan, Jianying Yan, Mei Xiao, Ying Li, Shihong Cui, Xianlan Zhao, Shangrong Fan, Ling Feng, Meihua Zhang, Yuyan Ma, Zishan You, Haixia Meng, Haiwei Liu, Jingxia Sun, Yan Cai, Kejia Hu, Huixia Yang

**Affiliations:** 1 Department of Obstetrics and Gynecology, Peking University First Hospital, Beijing 100034, China; juanjuan@bjmu.edu.cn (J.J.); weiyumei1982@126.com (Y.W.); elf-sg@126.com (G.S.); surina126014@126.com (R.S.); 2 Tianjin Central Obstetrics and Gynecology Hospital, Tianjin 300052, China; chenxu2665@126.com; 3 Jinan Maternity and Child Care Hospital, Jinan 250000, China; rqshan198@163.com; 4 Fujian Maternity and Child Health Hospital, Fuzhou 350001, China; yanjy2004@126.com; 5 Maternal and Child Hospital of Hubei Province, Wuhan 430070, China; xiaomei@hbfy.com; 6 Dalian Maternity Hospital, Dalian 116033, China; dlli.ying@163.com; 7 The Third Affiliated Hospital of Zhengzhou University, Zhengzhou 450052, China; shihongcui@126.com; 8 The First Affiliated Hospital of Zhengzhou University, Zhengzhou 450099, China; 13623812129@163.com; 9 Peking University Shenzhen Hospital, Shenzhen 518036, China; fanshangrong@163.com; 10 Tongji Hospital Affiliated to Huazhong University of Science and Technology, Wuhan 430030, China; fltj007@163.com; 11 Taiyuan Maternal and Child Health Hospital, Taiyuan 030012, China; zhangmeihua121@sina.com; 12 Qilu Hospital of Shandong University, Jinan 250012, China; yuyanma65@163.com; 13 Suzhou Jiulong Hospital Affiliated to Shanghai Jiaotong University, Suzhou 320571, China; youzishan@icloud.com; 14 Affiliated Hospital of Inner Mongolia Medical University, Hohhot 010050, China; mhx9097@163.com; 15 Hainan General Hospital, Haikou 570311, China; 15208981757@163.com; 16 The First Affiliated Hospital of Harbin Medical University, Harbin 150007, China; sjxsw@163.com; 17 The Fourth Affiliated Hospital of Harbin Medical University, Harbin 150026, China; caiyan318@126.com; 18 The Hospital of Shunyi District Beijing, Beijing 101300, China; 15300173210@163.com

**Keywords:** macrosomia, multipara, risk factor, multi-center retrospective study

## Abstract

The increased incidence of macrosomia has caused an enormous burden after the transition from the almost 40-year one-child policy to the universal two-child policy in 2015 and further to the three-child policy in 2021 in China. However, studies on risk factors of macrosomia in multipara under the new fertility policy in China are limited. We aim to explore the incidence and risk factors for macrosomia in multipara to provide the scientific basis for preventing macrosomia in multipara. A multi-center retrospective study was conducted among 6200 women who had two consecutive deliveries in the same hospital and their second newborn was delivered from January to October 2018 at one of 18 hospitals in 12 provinces in China. Macrosomia was defined as birth weight ≥ 4000 g. Logistic regression models were performed to analyze risk factors for macrosomia in multipara. The incidence of macrosomia in multipara was 7.6% (470/6200) and the recurrence rate of macrosomia in multipara was 27.2% (121/445). After adjusting for potential confounders, a higher prepregnancy BMI, higher gestational weight gain, history of macrosomia, a longer gestation in the subsequent pregnancy were independent risk factors of macrosomia in multipara (*p* < 0.05). Healthcare education and preconception consultation should be conducted for multipara patients with a history of macrosomia to promote maintaining optimal prepregnancy BMI and avoid excessive gestational weight gain to prevent macrosomia.

## 1. Introduction

Macrosomia is one of the most common adverse outcomes of newborns, which usually refers to the birthweight of a newborn ≥4000 g. Macrosomia complications include a high risk of shoulder dystocia, cesarean section, birth injury, asphyxia, postpartum hemorrhage, and perinatal death, and macrosomia newborns are more susceptible to metabolic disorders, such as obesity, type 2 diabetes mellitus, hypertension, etc. in later life [1,2,3]. The incidence of macrosomia has raised rapidly during the past several decades worldwide [4]. Data from the National Center for Health Statistics showed that macrosomia occurred in 7.8% of liveborn infants in the United States in 2018 [5]. A retrospective cohort study conducted in the UK between January 2009 and December 2016 showed that the incidence of fetal macrosomia was 12.7% [3]. There has also been a significant increase in the incidence of macrosomia in China due to economic development and living standard improvement, as well as lifestyle changes in dietary patterns and physical activity [6,7,8,9]. The increased incidence of macrosomia has caused an enormous social economic and health burden in China, especially after the transition from the almost 40-year one-child policy to the universal two-child policy in 2015 and further to the three-child policy in 2021. Multiparas are more likely to deliver macrosomia newborns as conventionally believed. However, the almost 40-year one-child policy in China made it hard to explore the risk factors for macrosomia in Chinese multipara and large studies on macrosomia in multipara after the transition from the one-child policy to the universal two-child policy in 2015 and further to three-child policy in 2021 in China are scarce. Less attention has been paid to risk factors for macrosomia in multipara. Early identification of risk factors for macrosomia in multipara could help to promote preventive measures to improve perinatal outcomes. Therefore, we aim to explore the incidence and risk factors of macrosomia in multipara in a multi-center retrospective study in China, as well as to further conduct stratified analysis according to different characteristics to provide a scientific basis for the prevention of macrosomia in multipara in China, which will be beneficial to guide appropriate clinical practice and avoid adverse perinatal outcomes.

## 2. Materials and Methods

### 2.1. Study Participants and Data Collection

A multi-center retrospective study was conducted among 18 hospitals in 12 provinces in China. Women of reproductive age who had two consecutive deliveries in the same hospital and the second newborn was delivered between January 2018 to October 2018 were included in our study. Women whose status of macrosomia was unknown were excluded from the current study.

Data of maternal age, prepregnancy weight, height, gestational weight gain, pregnancy complications, delivery mode, gestational age at delivery, birth weight, pregnancy outcomes in the first and subsequent pregnancy, inter-pregnancy interval, inter-pregnancy weight change, as well as history of disease of the participants were collected by consulting medical records. The primary outcome was macrosomia in multipara.

Macrosomia was defined as equal to or above 4000 g, irrespective of the gestational age. Prepregnancy body mass index (BMI) was calculated as maternal prepregnancy weight in kilograms divided by height in meters squared (kg/m^2^). Overweight and obesity were classified based on BMI recommendations of the Group of China Obesity Task Force of the Chinese Ministry of Health including underweight (BMI < 18.5 kg/m^2^), normal weight (18.5 ≤ BMI < 24.0 kg/m^2^), overweight (24.0 ≤ BMI < 28.0 kg/m^2^), and obesity (BMI ≥ 28.0 kg/m^2^) [10]. Adequacy of weight gain during pregnancy was defined according to the Chinese Nutrition Society’s recommendations based on maternal prepregnancy BMI status: a gain of 11.0–16.0 kg for underweight women; 8.0–14.0 kg for normal-weight women; 7.0–11.0 kg for overweight women, and; 5.0–9.0 kg for obese women [11]. Gestational diabetes mellitus (GDM) was diagnosed according to the 75 g oral glucose tolerance test (OGTT) during gestational weeks 24–28 for all pregnant women as universal screening. When one or more plasma glucose values equal to or above 5.1 mmol/L, 10.0 mmol/L, and 8.5 mmol/L at fasting, 1-h, and 2-h after the 75 g OGTT, the diagnosis could be made [12]. Gestational hypertension was defined as newly-onset hypertension, systolic blood pressure (SBP) ≥ 140 mmHg and/or diastolic blood pressure (DBP) ≥ 90 mmHg, starting after 20 weeks without proteinuria. Preeclampsia was defined as SBP ≥ 140 mmHg and/or DBP ≥ 90 mmHg on at least two occasions 4 h apart, developing after gestational week 20 in women with previously normal blood pressure, accompanied by proteinuria, or without proteinuria, newly-onset hypertension with a recent occurrence of any following symptoms: thrombocytopenia, impaired liver function, renal insufficiency, pulmonary edema, and cerebral or visual symptoms according to the recommendations of the American College of Obstetricians and Gynecologists (ACOG) [13].

### 2.2. Statistical Analysis

Characteristics of the study participants were summarized as mean ± standard deviation (SD) for continuous variables and numbers (percentages) for categorical variables. The *t*-test or χ^2^ test was performed to compare the differences between multipara who delivered macrosomia and non-macrosomia for continuous variables and categorical variables, respectively. Univariate and multivariate logistic regression models were performed with macrosomia in multipara as the outcome and risk factors as the exposure to obtain odds ratios (OR) and 95% confidence intervals (95% CI) to analyze the risk factors of macrosomia in multipara. Various confounding factors, including maternal age, prepregnancy BMI, gestational weight gain, GDM, gestational hypertension, preeclampsia, delivery gestational week in the first and subsequent pregnancy, as well as a history of macrosomia were adjusted for the multivariate models. Stratified analysis was further carried out based on the history of macrosomia, prepregnancy BMI, and the gestational weight gain in the subsequent pregnancy to explore the differences among women with different characteristics. All statistical analyses were carried out with SPSS 20.0 software. Two-sided *p* < 0.05 was considered statistically significant.

## 3. Results

A total of 6200 women with two consecutive deliveries in the same hospital from 18 hospitals in 12 provinces in China were included in our study. Overall, the incidence of macrosomia in multipara was 7.6% (470/6200) and the recurrence rate of macrosomia in multipara was 27.2% (121/445). The incidence of macrosomia in multipara without a history of macrosomia was 6.1% (349/5755).

The characteristics of study participants based on macrosomia in multipara are shown in Table 1. Among them, 470 multiparas (7.6%) delivered macrosomia, and 5730 multiparas (92.4%) delivered non-macrosomia. Multipara who delivered macrosomia as compared to multipara without delivering macrosomia showed higher prepregnancy BMI, gestational weight gain, and a longer gestation in both the first and subsequent pregnancies (*p* < 0.05). In addition, the rates of prepregnancy overweight and obesity, excessive gestational weight gain, GDM in both the first and subsequent pregnancy, as well as a history of macrosomia were significantly higher among multipara who delivered macrosomia compared to those without macrosomia (*p* < 0.05). There was no significant difference in maternal age, rates of hypertensive disorders of pregnancy in both first and subsequent pregnancy, inter-pregnancy interval, as well as inter-pregnancy weight change among multipara who delivered macrosomia and non-macrosomia (*p* > 0.05).

### 3.1. Risk Factors for Macrosomia in Multipara

The effects of risk factors on macrosomia in multipara are shown in Table 2. Higher prepregnancy BMI, gestational weight gain, GDM complications, longer gestation in the subsequent pregnancy, and history of macrosomia and GDM, as well as higher prepregnancy BMI and gestational weight gain in the first pregnancy, were associated with an increased risk of macrosomia in multipara (*p* < 0.05). After adjusting for potential confounding factors, only higher prepregnancy BMI and gestational weight gain, longer gestation in the subsequent pregnancy, as well as a history of macrosomia were still independently associated with the occurrence of macrosomia in multipara (*p* < 0.05). Multipara with a history of macrosomia had more than 5 times higher risk of delivering macrosomia in the subsequent pregnancy than those without a history of macrosomia (OR = 5.15, 95% CI: 3.78–7.02, *p* < 0.001). In addition, each 1 kg/m^2^ increment in prepregnancy BMI, each 1 kg increment in gestational weight gain, each 1 week increment in gestational age at delivery in the subsequent pregnancy were associated with an 11% (OR = 1.11, 95% CI: 1.04–1.18, *p* = 0.001), 8% (OR = 1.08, 95% CI: 1.05–1.11, *p* < 0.001), and 65% (OR = 1.65, 95% CI: 1.47–1.85, *p* < 0.001) increase in the risk of macrosomia in multipara, respectively.

### 3.2. Risk Factors for Macrosomia in Multipara According to History of Macrosomia

Stratified analysis was carried out based on the history of macrosomia (Table 3). After adjusting for potential confounding factors, both higher weight gain during pregnancy and longer gestational age at delivery significantly increased the risk of macrosomia in multipara with and without a history of macrosomia (*p* < 0.05). In addition, for multipara without a history of macrosomia, a higher prepregnancy BMI in the subsequent pregnancy could increase the risk of macrosomia by 13% (OR = 1.13, 95% CI: 1.09–1.17, *p* < 0.001).

### 3.3. Risk Factors for Macrosomia in Multipara According to Prepregnancy BMI

Stratified analysis was also carried out based on prepregnancy BMI in the subsequent pregnancy (Table 4). The risk factors of macrosomia in multipara with different prepregnancy BMI in the subsequent pregnancy were slightly different. After adjusting for potential confounding factors, for underweight multipara, a higher gestational weight gain, GDM complications, and a history of macrosomia significantly increased the risk of macrosomia in multipara (*p* < 0.05). For multipara with normal prepregnancy BMI, women with a history of macrosomia had more than 5 times higher risk of delivering macrosomia in multipara than those without a history of macrosomia (OR = 5.87, 95% CI: 4.01–8.60, *p* < 0.001). In addition, higher weight gain during pregnancy, longer gestation in the subsequent pregnancy, and higher prepregnancy BMI in the first pregnancy could also increase the risk of macrosomia in multipara by 8% (OR = 1.08, 95% CI: 1.05–1.12, *p* < 0.001), 63% (OR = 1.63, 95% CI: 1.42–1.88, *p* < 0.001), and 9% (OR = 1.09, 95% CI: 1.02–1.17, *p* = 0.02), respectively. For prepregnancy overweight multipara, higher weight gain during pregnancy (*p* = 0.008), GDM complications (*p* = 0.03), longer gestation (*p* < 0.001) in the subsequent pregnancy, and history of macrosomia (*p* < 0.001) were significantly associated with an increased risk of macrosomia in multipara. For obesity multipara, a history of macrosomia, a history of GDM, and the delivery gestational week in the subsequent pregnancy were independent risk factors of macrosomia in multipara (*p* < 0.05).

### 3.4. Risk Factors for Macrosomia in Multipara According to Weight Gain during Pregnancy

After stratified anaylsis based on weight gain during the subsequent pregnancy, a history of macrosomia was significantly associated with an increased risk of macrosomia in multiparas with inadequate, adequate, and excessive weight gain during pregnancy in the subsequent pregnancy in multivariable models (*p* < 0.001). Apart from a history of macrosomia, both a higher prepregnancy BMI and a longer gestation in the subsequent pregnancy were independent risk factors of macrosomia in multipara with adequate and excessive weight gain during the subsequent pregnancy (*p* < 0.05). History of GDM might increase the risk of delivering macrosomia in the subsequent pregnancy by 73% for multipara with excessive gestational weight gain in the subsequent pregnancy (OR = 1.73, 95% CI: 1.15–2.59, *p* = 0.009) (Table 5).

## 4. Discussion

In our study, we found that the incidence of macrosomia in multipara was 7.6% (470/6200) and the recurrence rate of macrosomia in multipara was 27.2% (121/445). The incidence of macrosomia in multipara without a history of macrosomia was 6.1% (349/5755). After adjusting for potential confounding factors, higher prepregnancy BMI, higher gestational weight gain, longer gestation in the subsequent pregnancy, as well as a history of macrosomia were independently associated with an increased risk of macrosomia in multipara. The risk factors of macrosomia in women with different prepregnancy BMIs, weight gain during the subsequent pregnancy, as well as a history of macrosomia were slightly different.

An increase in the incidence of macrosomia has been demonstrated worldwide, including in China [6,7,14,15]. According to previous studies, the incidence of macrosomia has risen from 6.0% in 1995 to 7.8% in 2005 in southeast China [6]. Studies in two hospitals in urban Beijing reported that the incidence of macrosomia has risen from 6.6% in 1996 to 7.0% in 2010 [7]. Similar to our results (7.6%), a hospital-based cross-sectional study of 14 provinces in China demonstrated that the prevalence of macrosomia was 7.3% [15].

Multipara with a history of macrosomia had a higher risk of delivering another macrosomia in the subsequent pregnancy [16]. Mahony et al. reported that 32% of women who delivered first-pregnancy macrosomia might deliver second-pregnancy macrosomia [17]. We found that the recurrence rate of macrosomia in multipara was 27.2%, which was in accordance with a study conducted by Fang et al. with a recurrence rate of 23.2% [18]. Macrosomia recurrence is of growing concern under the new fertility policy in China as most of the risk factors of macrosomia persist or become even worse in the subsequent pregnancy, and history of macrosomia is one of the most significant risk factors for macrosomia in multipara. Moreover, as illustrated by previous studies, the maternal peritoneal and uterine wall of multiparas is more relaxed than that of primiparas, which might cause an increase in uterine volume, thus leading to an increased risk of fetus macrosomia [14,19,20]. Therefore, preventing macrosomia from the first pregnancy is of great importance to avoid macrosomia in multipara.

We found that apart from a history of macrosomia, a higher prepregnancy BMI and gestational weight gain in the subsequent pregnancy were independent risk factors for macrosomia in multipara, which was consistent with previous studies [21,22,23,24]. A previous cohort study of 105,768 mother-child pairs [25] and a 10-year cross-sectional study of 84,900 participants [26] have reported that maternal low BMI was inversely associated with macrosomia. However, most of the previous studies were conducted on the European and American populations rather than the Chinese population. It is well-known that the classification of obesity and overweight, as well as recommendations for gestational weight gain, were different between China and other countries. According to the WHO, a BMI of more than 25.0 kg/m^2^ and 30.0 kg/m^2^ were defined as overweight and obese respectively for European and American whites [27]. However, this classification was not necessarily applicable for Asians as the upper limit of the normal range of 24.9 kg/m^2^ was too high for Asians. Experts believed that a BMI greater than 24.0 kg/m^2^ and 28.0 kg/m^2^ should be used to define overweight and obese for the Chinese population [10]. For gestational weight gain, the Institute of Medicine (IOM) recommended a gain of 12.5–18.0 kg for underweight women; 11.5–16.0 kg for normal-weight women; 7.0–11.5 kg for overweight women, and; 5.0–9.0 kg for obese women [28]. As the Chinese BMI classification was different, in October 2021, a new recommendation for gestational weight gain for the Chinese population was issued by the Chinese Nutrition Society based on the Chinese maternal prepregnancy BMI status, including: a gain of 11.0–16.0 kg for underweight women (BMI < 18.5 kg/m^2^); 8.0–14.0 kg for normal-weight women (18.5 ≤ BMI < 24.0 kg/m^2^); 7.0–11.0 kg for overweight women (24.0 ≤ BMI < 28.0 kg/m^2^), and; 5.0–9.0 kg for obese women (BMI ≥ 28.0 kg/m^2^) [11]. In addition, studies that explore these risk factors for macrosomia in Chinese multipara are limited. Therefore, there is an urgent need to research this in Chinese multipara using China’s own classification of BMI and recommendations for gestational weight gain under the new fertility policy. Clinically, identifying multipara who are more likely to deliver macrosomia might be important in planning future pregnancies and preconception counseling for high-risk women and it has been reported that prepregnancy BMI and gestational weight gain were risk factors of macrosomia that were most amenable to intervention, and had potential health benefits beyond pregnancy and childbirth. Therefore, women should maintain their prepregnancy BMI in a normal range before planning to fall pregnant again [18]. In addition, more intensive behavioral and dietary interventions, together with weight gain control and monitoring, might be needed in high-risk multipara to minimize the risk of macrosomia.

Studies have demonstrated that pregnant women complicated with GDM are more likely to deliver macrosomia [29,30]. However, in the current study, we demonstrated that GDM was not significantly associated with the risk of macrosomia in multipara, which might be due to the One-Day Care Clinic for pregnancies with GDM in our study centers. All pregnant women diagnosed with GDM went to the One-Day Care Clinic to be educated on the basic knowledge of GDM, medical nutrition therapy, physical exercise, weight management, and blood glucose self-monitoring methods by professional physicians, nurses, and clinical nutritionists. In addition, the gestational weight gain targets were stricter for GDM participants [12]. As reported by previous studies, medical nutrition therapy to control GDM could decrease macrosomia by 73% in GDM women [31] and for diabetic women with good glycemic control, the rate of macrosomia approaches that of the general population [32]. In addition, ethnicity might also play a role in the non-significant association between GDM and macrosomia in multipara. According to a case-control study of five ethnic groups, GDM was an independent predictor of macrosomia in South-Central Asian, Latin-American, and Moroccan but not in East-Asian or Caucasian women [33]. Thus, prevention and management of GDM as well as closely monitoring and controlling for glycemia during pregnancy should be suggested to prevent the occurrence of macrosomia.

This is a relatively large multi-center study with participants of two consecutive deliveries that took place in the same hospital under the new fertility policy in China. Even though the risk factors for macrosomia in multipara reported by our study were similar to previous studies, the study was worthy to conduct as (1) the previous studies were mostly conducted in European and American populations rather than the Chinese population and the prevalence and risk factors for macrosomia in different races are different; (2) previous studies were not specified to multipara in the Chinese population as the almost 40-year one-child policy in China made it hard to explore the risk factors and prevalence of macrosomia in Chinese multipara, and there is an urgent need to explore this in Chinese multipara after the transition from the one-child policy to the universal two-child policy in 2015 and further to the three-child policy in 2021 enacted by the Chinese government; (3) we collected information of the multipara in both the first and subsequent pregnancy to make the analysis more comprehensive, and; (4) though the risk factors for macrosomia in multipara reported by our study were similar to previous studies, the results were new under the new fertility policy in China and provided a scientific basis and important implications for understanding and preventing macrosomia in Chinese multipara in the new fertility policy era. Our findings are useful to inform policy-makers regarding preconception consultation and healthcare education for multipara to prevent delivering macrosomia, as well as to promote maternal and child health. However, several limitations should be taken into consideration. First, the current study was limited by its retrospective design. In addition, as the information was extracted from medical records, data on certain potential confounding factors, including lifestyle characteristics during pregnancy, such as diet and physical exercise were not available. So, we could not exclude these potential confounding effects. Moreover, the sample size might not be large enough for stratification analysis, such as for the prepregnancy obesity group and inadequate gestational weight gain group, which may lack power for a robust assessment. More high-quality prospective studies with a larger sample size are needed in the future.

## 5. Conclusions

The increasing incidence and recurrence of macrosomia have become an enormous burden under the new fertility policy in China. Higher prepregnancy BMI and weight gain during pregnancy are independently associated with an increased risk of macrosomia in multipara. Moderating weight before pregnancy and optimizing weight gain during pregnancy are modifiable factors to reduce macrosomia in multipara. Healthcare education and consultation should be conducted for multipara to promote maintaining an optimal prepregnancy BMI and avoid excessive gestational weight gain in order to prevent macrosomia in multipara. In addition, a history of macrosomia could largely increase the risk of macrosomia in multipara. Thus, multipara with a history of macrosomia should be encouraged to have preconception counseling before preparing for a subsequent pregnancy. It is of great significance for preventing macrosomia and improving maternal and neonatal health in multipara in China.

## Figures and Tables

**Table 1:** Characteristics of the study participants according to macrosomia in multipara.

	Macrosomia (n = 470)	Non-Macrosomia (n = 5730)	t/χ^2^	*p*
Maternal age (years)	31.92 ± 3.35	31.91 ± 3.40	−0.091	0.928
First pregnancy				
Prepregnancy body mass index (kg/m^2^)	22.42 ± 3.30	21.52 ± 3.08	−5.444	<0.001
Underweight: <18.5	28 (7.4%)	702 (14.6%)	25.112	<0.001
Normal weight: 18.5–23.9	246 (65.4%)	3185 (66.4%)		
Overweight: 24.0–27.9	86 (22.9%)	745 (15.5%)		
Obesity: ≥28.0	16 (4.3%)	167 (3.5%)		
Weight gain during pregnancy (kg)	15.64 ± 5.58	15.04 ± 5.46	−2.011	0.044
Inadequate	48 (13.3%)	876 (18.6%)	15.160	0.001
Adequate	133 (36.3%)	1923 (40.8%)		
Excessive	185 (50.5%)	1920 (40.7%)		
Gestational diabetes mellitus	95 (20.2%)	906 (15.8%)	6.215	0.013
Hypertensive disorders of pregnancy	13 (2.8%)	151 (2.6%)	0.029	0.865
Macrosomia	121(26.0%)	324(5.8%)	259.374	<0.001
Gestational age at delivery (weeks)	39.31 ± 1.41	39.07 ± 1.56	−3.215	0.001
Subsequent pregnancy				
Prepregnancy body mass index (kg/m^2^)	23.33 ± 3.62	22.17 ± 3.21	−6.721	<0.001
Underweight: <18.5	16 (4.2%)	479 (10.1%)	34.499	<0.001
Normal weight: 18.5–23.9	225 (59.2%)	3101 (65.2%)		
Overweight: 24.0–27.9	106 (27.9%)	930 (19.6%)		
Obesity: ≥28.0	33 (8.7%)	247 (5.2%)		
Weight gain during pregnancy (kg)	14.57 ± 5.29	13.44 ± 4.86	−3.791	<0.001
Inadequate	44 (13.1%)	1072 (25.5%)	43.777	<0.001
Adequate	133 (39.6%)	1807 (42.9%)		
Excessive	159 (47.3%)	1329 (31.6%)		
Gestational diabetes mellitus	120 (25.5%)	1207 (21.1%)	7.105	0.029
Hypertensive disorders of pregnancy	14 (3.0%)	135 (2.4%)	0.718	0.397
Gestational age at delivery (weeks)	39.32 ± 1.03	38.61 ± 1.43	−13.789	<0.001
Inter-pregnancy				
Inter-pregnancy interval (years)	3.48 ± 1.37	3.51 ± 1.37	0.399	0.69
Inter-pregnancy weight change (kg)	2.24 ± 6.28	1.64 ± 6.01	−1.813	0.07

**Table 2:** Effects of risk factors on macrosomia in multipara.

	Model 1 *		Model 2 ^†^		Model 3 ^‡^	
	OR (95% CI)	*p*	OR (95% CI)	*p*	OR (95% CI)	*p*
Maternal age (years)	1.00 (0.97,1.03)	0.93	1.03 (0.99,1.07)	0.12	1.03 (0.99,1.07)	0.12
Prepregnancy body mass index (kg/m^2^)	1.10 (1.07,1.13)	<0.001	1.13 (1.10,1.17)	<0.001	1.11 (1.04,1.18)	0.001
Weight gain during pregnancy (kg)	1.05 (1.02,1.07)	<0.001	1.08 (1.05,1.10)	<0.001	1.08 (1.05,1.11)	<0.001
Gestational diabetes mellitus	1.31 (1.07,1.60)	0.009	1.38 (1.07,1.78)	0.01	1.10 (0.82,1.46)	0.53
Gestational hypertension	1.27 (0.73,2.22)	0.40	1.33 (0.63,2.78)	0.45	1.35 (0.59,3.07)	0.48
Preeclampsia	0.41 (0.10,1.69)	0.22	0.25 (0.03,1.98)	0.19	0.27 (0.03,2.34)	0.24
Delivery gestational week (weeks)	1.65 (1.51,1.81)	<0.001	1.62 (1.46,1.81)	<0.001	1.65 (1.47,1.85)	<0.001
History of macrosomia	5.74 (4.53,7.26)	<0.001	5.36 (3.99,7.20)	<0.001	5.15 (3.78,7.02)	<0.001
Prepregnancy body mass index in the first pregnancy (kg/m^2^)	1.09 (1.06,1.12)	<0.001	1.04 (0.99,1.10)	0.15	1.00 (0.94,1.07)	0.92
Weight gain during pregnancy in the first pregnancy (kg)	1.02 (1.00,1.04)	0.044	0.99 (0.97,1.02)	0.62	0.99 (0.96,1.01)	0.36
History of gestational diabetes mellitus	1.35 (1.07,1.71)	0.01	1.34 (0.99,1.81)	0.05	1.33 (0.96,1.83)	0.09
History of gestational hypertension	1.05 (0.59,1.87)	0.87	1.22 (0.63,2.39)	0.56	1.67 (0.79,3.54)	0.18
History of preeclampsia	0.45 (0.17,1.23)	0.12	0.73 (0.26,2.05)	0.55	0.52 (0.14,1.86)	0.31
Interpregnancy interval (years)	0.99 (0.92,1.06)	0.69	1.06 (0.97,1.16)	0.19	1.04 (0.94,1.15)	0.43
Interpregnancy weight change (kg)	1.02 (0.99,1.03)	0.07	0.99 (0.97,1.01)	0.25	1.15 (0.97,1.37)	0.11

* Model 1 without any adjustment; ^†^ Model 2 mutually adjusted for maternal age, prepregnancy body mass index, gestational weight gain, gestational diabetes mellitus, gestational hypertension, preeclampsia, delivery gestational week in subsequent pregnancy; ^‡^ Model 3 mutually adjusted for maternal age, prepregnancy body mass index, gestational weight gain, gestational diabetes mellitus, gestational hypertension, preeclampsia, delivery gestational week in the subsequent pregnancy, prepregnancy body mass index, gestational weight gain in the first pregnancy, history of macrosomia, gestational diabetes mellitus, gestational hypertension, and preeclampsia.

**Table 3:** Effects of risk factors on macrosomia in multipara according to the history of macrosomia.

History of Macrosomia	With History of Macrosomia		Without History of Macrosomia	
	OR (95% CI) *	*p*	OR (95% CI) *	*p*
Maternal age (years)	1.02 (0.94,1.10)	0.71	1.04 (0.99,1.08)	0.08
Prepregnancy body mass index (kg/m^2^)	1.08 (0.99,1.17)	0.08	1.13 (1.09,1.17)	<0.001
Gestational weight gain (kg)	1.06 (1.00,1.12)	0.04	1.08 (1.05,1.11)	<0.001
Gestational diabetes mellitus	0.93 (0.56,1.57)	0.80	1.33 (0.98,1.80)	0.06
Delivery gestational week (weeks)	1.54 (1.23,1.94)	<0.001	1.69 (1.49,1.91)	<0.001
Prepregnancy body mass index in first pregnancy (kg/m^2^)	1.01 (0.90,1.14)	0.85	1.03 (0.96,1.10)	0.40
Gestational weight gain in first pregnancy (kg)	1.03 (0.98,1.09)	0.24	0.97 (0.95,1.00)	0.05
History of gestational diabetes mellitus	1.33 (0.69,2.56)	0.40	1.39 (0.98,1.96)	0.07
Interpregnancy interval (years)	1.08 (0.87,1.34)	0.52	1.03 (0.92,1.14)	0.63
Interpregnancy weight change (kg)	1.00 (0.95,1.04)	0.88	0.99 (0.97,1.02)	0.52

* Adjusted for maternal age, prepregnancy body mass index, gestational weight gain, gestational diabetes mellitus, and delivery gestational week in the subsequent pregnancy.

**Table 4:** Effects of risk factors on macrosomia in multipara according to pre-pregnancy BMI.

Pre-Pregnancy Body Mass Index in Subsequent Pregnancy	Underweight		Normal Weight		Overweight		Obesity	
	OR (95% CI) *	*p*	OR (95% CI) *	*p*	OR (95% CI) *	*p*	OR (95% CI) *	*p*
Maternal age (years)	0.87 (0.73,1.05)	0.14	1.04 (0.99,1.09)	0.09	1.07 (0.99,1.15)	0.07	0.98 (0.86,1.11)	0.74
Gestational weight gain (kg)	1.11 (1.01,1.21)	0.02	1.08 (1.05,1.12)	<0.001	1.07 (1.02,1.13)	0.008	0.98 (0.90,1.07)	0.71
Gestational diabetes mellitus	4.80 (1.42,16.20)	0.01	0.98 (0.67,1.42)	0.90	1.68 (1.06,2.65)	0.03	0.96 (0.41,2.23)	0.92
Delivery gestational week (weeks)	1.41 (0.92,2.17)	0.12	1.63 (1.42,1.88)	<0.001	1.73 (1.39,2.15)	<0.001	1.68 (1.15,2.45)	0.007
History of macrosomia	9.15 (1.94,43.19)	0.005	5.87 (4.01,8.60)	<0.001	5.39 (3.12,9.28)	<0.001	2.81 (1.01,7.79)	0.047
Prepregnancy body mass index in first pregnancy (kg/m^2^)	0.87 (0.65,1.17)	0.36	1.09 (1.02,1.17)	0.02	1.02 (0.93,1.12)	0.71	1.11 (0.98,1.27)	0.11
Gestational weight gain during pregnancy in first pregnancy (kg)	1.02 (0.90,1.15)	0.76	0.97 (0.93,1.00)	0.05	1.01 (0.96,1.05)	0.81	1.02 (0.94,1.11)	0.63
History of gestational diabetes mellitus	2.38 (0.58.9.80)	0.23	1.19 (0.77,1.85)	0.44	1.23 (0.71,2.15)	0.46	3.49 (1.35,9.01)	0.01
Interpregnancy interval (years)	0.87 (0.53,1.42)	0.57	1.09 (0.97,1.23)	0.15	0.93 (0.77,1.12)	0.43	1.08 (0.77,1.52)	0.65
Interpregnancy weight change (kg)	1.10 (0.97,1.24)	0.16	0.99 (0.96,1.02)	0.56	0.99 (0.95,1.03)	0.67	1.03 (0.98,1.08)	0.26

* Adjusted for maternal age, gestational weight gain, gestational diabetes mellitus, delivery gestational week in the subsequent pregnancy, and history of macrosomia.

**Table 5:** Effects of risk factors on macrosomia in multipara according to gestational weight gain.

Gestational Weight Gain in Subsequent Pregnancy	Inadequate		Adequate		Excessive	
	OR (95% CI) *	*p*	OR (95% CI) *	*p*	OR (95% CI) *	*p*
Maternal age (years)	1.02 (0.87,1.20)	0.79	1.02 (0.97,1.09)	0.43	1.04 (0.99,1.09)	0.10
Prepregnancy body mass index (kg/m^2^)	1.03 (0.86,1.23)	0.77	1.12 (1.05,1.19)	<0.001	1.06 (1.05,1.14)	0.006
Gestational diabetes mellitus	0.69 (0.23,2.03)	0.50	1.19 (0.78,1.83)	0.42	1.25 (0.88,1.78)	0.21
Delivery gestational week (weeks)	1.61 (0.91,2.86)	0.10	1.68 (1.40,2.01)	<0.001	1.63 (1.42,1.88)	<0.001
History of macrosomia	15.94 (4.53,56.03)	<0.001	4.02 (2.39,6.78)	<0.001	5.73 (3.93,8.36)	<0.001
Prepregnancy body mass index in first pregnancy (kg/m^2^)	0.87 (0.64,1.17)	0.34	1.00 (0.90,1.11)	0.92	1.07 (0.99,1.14)	0.08
Gestational weight gain in first pregnancy (kg)	0.98 (0.87,1.11)	0.80	0.99 (0.95,1.04)	0.72	0.99 (0.96,1.02)	0.45
History of gestational diabetes mellitus	0.93 (0.22,3.90)	0.92	1.06 (0.64,1.77)	0.82	1.73 (1.15,2.59)	0.009
Interpregnancy interval (years)	1.11 (0.74,1.67)	0.61	0.99 (0.85,1.16)	0.92	1.07 (0.94,1.21)	0.30
Interpregnancy weight change (kg)	1.06 (0.95,1.19)	0.31	1.01 (0.97,1.05)	0.76	0.98 (0.95,1.00)	0.10

* Adjusted for maternal age, prepregnancy body mass index, gestational diabetes mellitus, delivery gestational week in the subsequent pregnancy, and history of macrosomia.

## Data Availability

The data presented in this study are available on request from the corresponding author.
